# Supplementary material for: The interactome of intact mitochondria by cross-linking mass spectrometry provides evidence for coexisting respiratory supercomplexes
Source: Mol Cell Proteomics. 2017 Dec 8;17(2):216–32. doi: 10.1074/mcp.RA117.000470 (PMC5795388; doi:10.1074/mcp.RA117.000470)
Supplement: Supplemental Data [file supp_RA117.000470_133922_0_supp_23978_fzffwf.zip › spectra_annotation/mito_spectra_annotation/7-1-14-1-11-1.pdf]

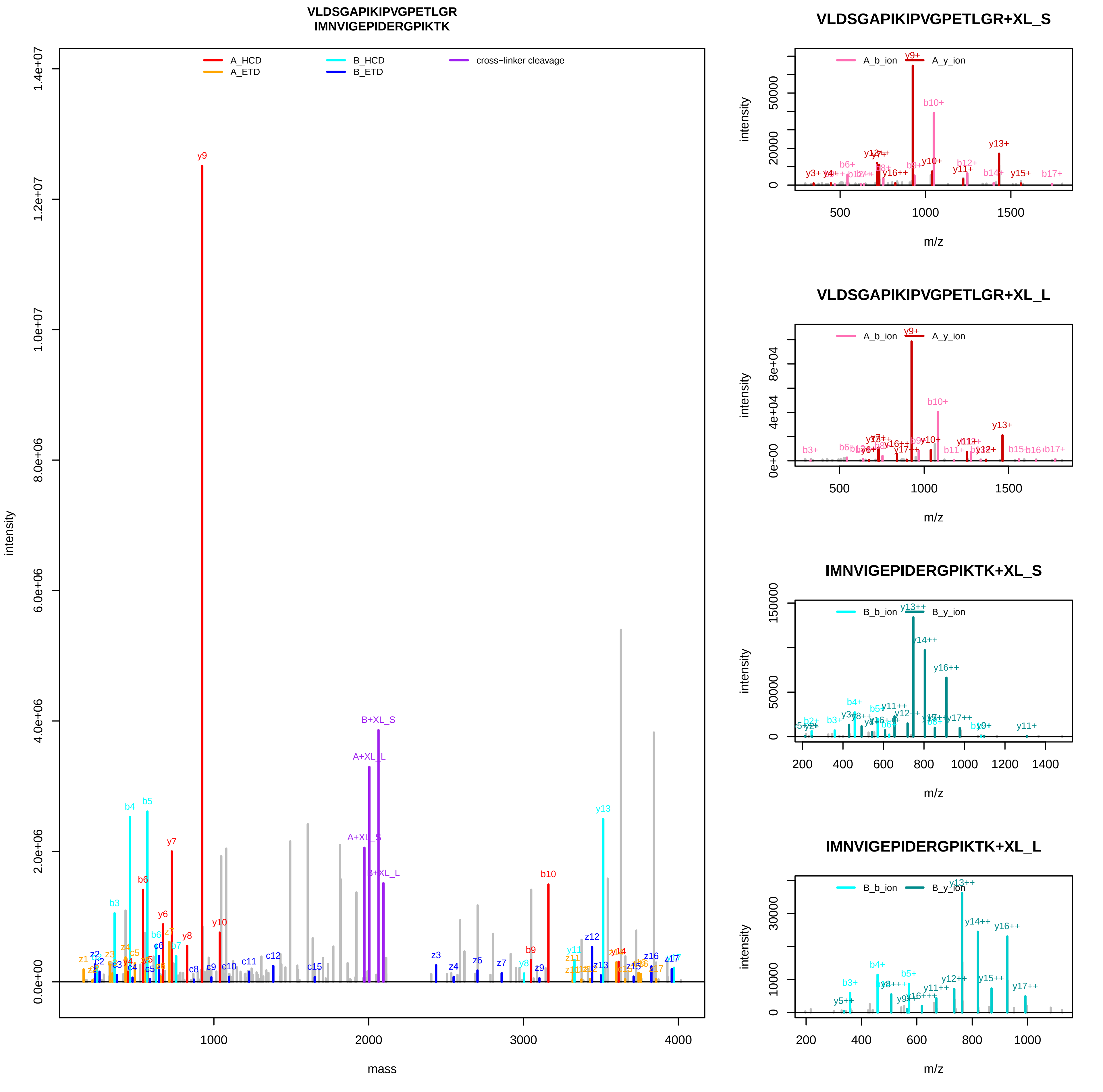
VLDSGAPIKIPVGPETLGR
IMNVIGEPIDERGPIKTK
A_HCD
A_ETD
B_HCD
B_ETD
cross−linker cleavage
intensity
mass
y9
y7
b6
y6
y10
y8
b4
b5
b3
B+XL_S
A+XL_L
A+XL_S
B+XL_L
y13
b10
b9
z12
z17
z16
c12
c11
c15
z3
z4
z6
z7
VLDSGAPIKIPVGPETLGR+XL_S
VLDSGAPIKIPVGPETLGR+XL_L
IMNVIGEPIDERGPIKTK+XL_S
IMNVIGEPIDERGPIKTK+XL_L
A_b_ion
A_y_ion
B_b_ion
B_y_ion
m/z
y9+
b10+
y13+
y13++
y14++
y16++
